# Risk factors for wound complications of closed calcaneal fractures after surgery: a systematic review and meta-analysis

**DOI:** 10.1186/s13049-015-0092-4

**Published:** 2015-02-08

**Authors:** Wei Zhang, Erman Chen, Deting Xue, Houfa Yin, Zhijun Pan

**Affiliations:** Department of Orthopedics, Second Affiliated Hospital, School of Medicine, Zhejiang University, Hangzhou, People’s Republic of China; Eye Center, Second Affiliated Hospital of Zhejiang University School of Medicine, Hangzhou, Zhejiang Province People’s Republic of China

**Keywords:** Calcaneus, Fracture, Complications, ORIF

## Abstract

**Background:**

To better clinical outcomes, open reduction and internal fixations (ORIFs) have been commonly performed in the case of closed displaced intra-articular calcaneal fractures (CDICFs). Nonetheless, postoperative wound complications remain a significant problem. Therefore, the aim of our study is to summarise relevant evidence investigating the risk factors for postoperative wound complications of CDICFs following ORIFs.

**Methods:**

A meta-analysis was conducted on relevant clinical studies to identify the risk factors for wound complications of CDICFs after ORIFs. Electronic databases were searched for all relevant studies up to October 2014. The Newcastle-Ottawa scale was used to evaluate the methodological quality, and study-specific odds ratios (ORs) were pooled using the fixed-effects model or random-effects model. Sensitivity analysis and meta-regression analysis was performed to evaluate the heterogeneity.

**Results:**

Ten observational studies involving 1559 patients with 1651 fractures were included in this meta-analysis. The results showed that diabetes (OR, 9.76; *p* < 0.01), no drainage (OR, 5.86; *p* < 0.01), fracture severity (OR, 3.31; *p* < 0.01) and bone graft (OR, 1.74; *p* < 0.01) were the risk factors for wound complications of CDICFs after ORIFs. A trend of more wound complications in patients with a history of smoking was detected. However, female patients, ORIFs performed within 14 days of injury, smoking, hypertension and drinking did not significantly increase the risk of wound complications (*p* > 0.05).

**Conclusions:**

Based on available relevant evidence, bone graft, diabetes, no drainage and fracture severity were all associated with an increased risk of wound complications after ORIF for CDICFs.

**Electronic supplementary material:**

The online version of this article (doi:10.1186/s13049-015-0092-4) contains supplementary material, which is available to authorized users.

## Background

Calcaneal fractures are one of the most common fractures of the hind foot [1]. They generally occur in the setting of high-energy trauma, often resulting in displaced intra-articular calcaneal fractures (CDICFs). Moreover, approximately 75% of patients with calcaneal fractures had CDICFs [2], which might continue to have devastating consequences for many patients. It also played a major socioeconomic impact with regard to time lost from work and recreation [3]. As the old saying goes, “the man who breaks his heel bone is done, so far as his industrial future is concerned” [4].

There has been a matter of conflict on the treatment of CDICFs. Open reduction and internal fixation (ORIF) would restore calcaneal height, the mechanical axis of the hindfoot and subtalar joint [5], which theoretically provides patients with the possibility of painless weight bearing in daily activities. Several clinical trials reported that patients with ORIF had better clinical outcomes, such as less pain, better clinical scores and later osteoarthritis [6-10]. However, the rapid growth of operative quantity coincided with a considerable rate of wound complications (2% to 27%) [11,12]. These complications would not only make patients suffer, but increase hospitalisations and expenses [13]. Worse, patients then require surgical debridement, hardware removal or myocutaneous flap coverage to eradicate infection.

Therefore, it is important to reduce the rate of wound complications when CDICFs were treated with ORIFs. To identify the risk factors for wound complications, several studies have been published [14-23], but some risk factors remains controversial. In order to provide a reference for clinical practice, it is necessary to have a meta-analysis to evaluate and summarize this issue. The aim of our study was to identify the risk factors for postoperative wound complications of CDICFs after ORIFs.

## Methods

This meta-analysis was performed according to the preferred reporting items for systematic reviews and meta-analyses guidelines (the PRISMA statement) (Additional file 1) [24].

### Retrieval strategies

Electronic databases, including PubMed, Embase and Cochrane library, were searched by three independent researchers (WZ, EMC and DTX), which were published up to October 10, 2014. The following keywords or corresponding Medical Subject Headings (MeSH) were used: “fractures of the os calcis”, “calcaneal fractures”, “fractures of calcaneum”, “fractures of calcaneus”, “fractures of hindfoot”, “complications”, “infection”, “debridement”, “oozing”. Full details of search strategy are included in Additional files 2 and 3. Meanwhile, reference lists of the relevant articles were also retrieved for any additional relevant studies. Languages were not restricted in the search.

### Inclusion criteria

We identified studies according to the following inclusion criteria: (1) participants: human adults (minimum of 18 years of age) with CDICFs; (2) intervention: ORIF; (3) comparison: patients with potential risk factors versus patients without potential risk factors resulting in higher or lower rate of wound complications; (4) sufficient data were available for estimating an odds ratio (OR) with confidence interval (CI).

The following criteria were used for exclusion: (1) minimally invasive reduction and fixation; (2) CDICFs with a primary arthrodesis; (3) animal studies and cadaver studies.

### Data extraction

Three authors (WZ, EMC and DTX) extracted relevant data independently, including the first author’s name, study region, study design, average age of participants, the number of case group (patients who experienced wound complications) and control group (patients who did not experience wound complications), operative approach, type of closure (one-layered or double-layered), duration of follow-up, potential risk factor for wound complications, and the number of major complications (including deep infection, osteomyelitis, and postoperative debridement involving hardware removal, free myocutaneous flap wound coverage and even amputation, etc.). In addition, we also tried to contact the authors of the eligible studies to ask for relevant original data for this meta-analysis.

### Quality assessment

In terms of the Newcastle-Ottawa Quality Assessment Scale [25], the methodological quality of each included study was assessed by two independent researchers (WZ and EMC). If disagreements were encountered, they were resolved by discussion with the corresponding author (ZJP). A maximum of nine points was assigned to each study: four for selection, two for comparability, and three for assessment of outcomes (for cohort studies) or exposures (for case-control studies).

### Statistical analysis

The statistical analyses were performed with Stata 12.0 software (StataCorp LP, College Station, Texas, USA). The ORs with corresponding 95% CIs were considered as the effect estimates for all included studies. Study-specific ORs were pooled using fixed-effects model or random-effects models. The statistical heterogeneity was assessed with the Q-test and *I*^*2*^*. I*^*2*^ > 50% was considered as high statistical heterogeneity [26]. If *p* > 0.1 and *I*^*2*^ < 50%, the fixed-effects model was used; otherwise, we used the random-effects model. *I*^*2*^ > 50% was considered as high heterogeneity. Then, sensitivity analysis was conducted by omitting one study at one time and examining the influence of each individual study on the overall OR. To identify the origin of the heterogeneity among studies, we conducted meta-regression analysis based on methodological quality or clinical diversity (study design, study region, type of closure, operative approach, etc.). Egger’s test and Begg’s test were performed to assess the publication bias. For all statistical analyses except heterogeneity and publication bias, a value of *p* < 0.05 was regarded as statistically significant, and all tests were two-sided.

## Results

### Study selection

**Figure 1:**
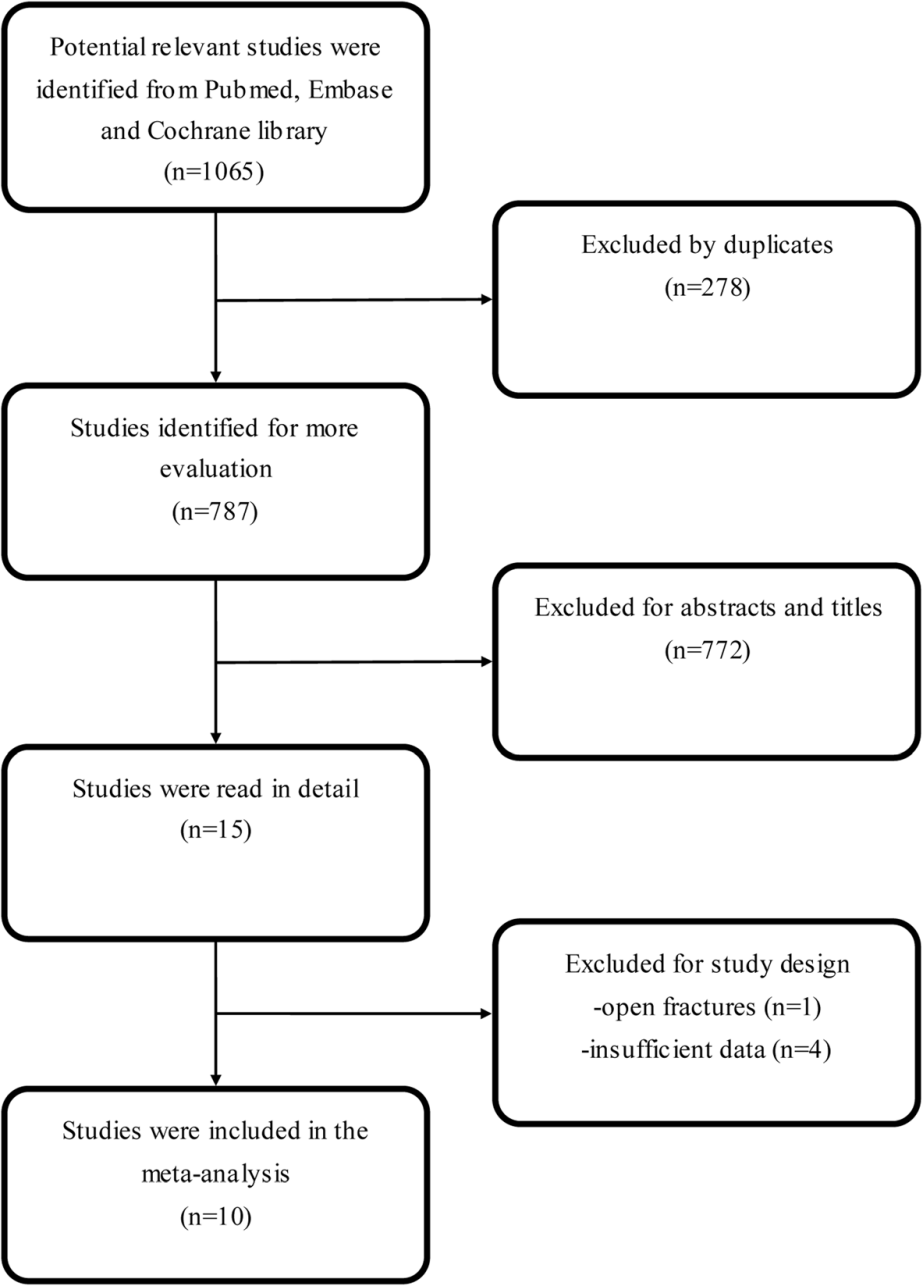
**Flow chart summarizing the selection process of studies.**

The process of study selection is presented in Figure 1. According to our search strategies, 1065 potential relevant articles were identified initially: 393 from PubMed, 649 from Embase and 23 from the Cochrane library. Of these, 278 studies were excluded as duplicates. After viewing the titles and abstracts of the 787 remaining studies, 15 studies were retrieved in full text. Among those, one study was excluded due to it involving open calcaneal fractures [27]. Sufficient data were not available in four studies, thus they were also excluded [13,28-30]. Finally, 10 observational studies were included in this meta-analysis [14-23]. There was an excellent interrater agreement between investigators on eligibility (Κ = 1.0).

### Study characteristics

The characteristics of the 10 included studies are presented in Table 1, of which eight studies was case-control studies and two were cohort studies. Among them, three were from Asia, five from Europe, one from America, and one from Oceania, respectively. The dataset involved 1559 patients with 1651 calcaneal fractures, of which 349 fractures were in the case group and 1302 fractures in the control group. The incidence of overall wound complications was 21.1%, including wound edge necrosis, haematoma, wound dehiscence or separation, erythema, infection, etc. The major complications (deep infection, osteomyelitis, surgical treatments including surgical irrigation and debridement, hardware removal, free myocutaneous flap wound coverage and even amputation, etc.) rate was 9.6%, which accounted for 46% of overall wound complications. The average age was 40.4 years. The standard L-shaped extensile lateral approach was used in six studies, the extended lateral approach was performed in two studies and the surgical approach of the remaining two studies was unavailable. Seven of 10 included studies performed double-layered closure. Follow-up periods ranged from 10.7 months to 48 months.

**Table 1:** **Study characteristics**

**Study**	**Publication year**	**Country**	**Study design**	**Group size (fractures)**		**Total patients (fractures)**	**Major complications (fractures)**	**Average age (years)**	**Operative approach**	**Closure method**	**Follow-up period (months)**
				**Case**	**Control**						
Wu [14]	2014	China	Case–control study	21	218	209 (239)	0	37.6	L-shaped lateral	2 layer	NA
Soni [15]	2014	UK	Retrospective cohort study	10	59	69 (69)	2	38.0	L-shaped lateral	2 layer	NA
Backes [22]	2014	Netherlands	Case-control study	57	134	191 (191)	19	NA	Extended lateral	1 layer/2 layer	>12.0
Ding [16]	2013	China	Case-control study	87	413	479 (490)	59	45.0	L-shaped lateral	2 layer	14.2
Hao [17]	2013	China	Case-control study	17	46	58 (63)	3	35.0	L-shaped lateral	2 layer	12.0
Court-Brown [21]	2009	UK	Case-control study	45	133	178 (178)	10	41.0	Extended lateral	NA	NA
Koski [18]	2005	Finland	Case-control study	35	113	126 (148)	20	39.8	L-shaped lateral	2 layer	10.7
Assous [23]	2001	UK	Retrospective cohort study	13	27	40 (40)	0	35.0	NA	NA	27.0
Al-Mudhaffar [19]	2000	UK	Case-control study	6	27	30 (33)	2	41.0	NA	2 layer	48.0
Folk [20]	1999	USA	Case-control study	58	132	179 (190)	40	35.0	L-shaped lateral	2 layer	NA
Total	--	--	--	349	1302	1559 (1651)	155	40.4	--	--	--

NA, not available; major complications included deep infection, osteomyelitis, and operative debridement, etc.

### Study quality

Table 2 shows the quality of included studies. The average score for the quality assessment of included studies was 7.10.

**Table 2:** **Study quality**

**Case–control study**	**Selection**				**Comparability**	**Exposure**			**Total quality score**
	**Adequate definition of cases**	**Representativeness of cases**	**Selection of controls**	**Definition of controls**	**Control for important factors or additional factors**	**Ascertainment of exposure (blinding)**	**Same method of ascertainment for subjects**	**Non-response rate**	
Wu [14]	1	1	1	1	1	1	1	1	8
Backes [22]	1	1	1	1	1	1	1	1	7
Ding [16]	1	1	1	1	1	1	1	1	8
Hao [17]	0	1	1	1	1	1	0	1	6
Court-Brown [21]	0	1	0	1	1	1	0	1	5
Koski [18]	1	1	1	1	1	1	0	1	7
Al-Mudhaffar [19]	1	1	1	1	1	1	1	1	8
Folk [20]	1	1	1	1	1	1	1	1	8
**Cohort study**	**Selection**				**Comparability**	**Outcome**			**Total quality score**
	**Representativeness of the exposed cohort**	**Selection of the non-exposed cohort**	**Ascertainment of exposure**	**Demonstration that outcome of interest was not present at start of study**	**Control for important factors or additional factors**	**Assessment of outcome**	**Follow-up was long enough for outcomes to occur**	**Adequacy of follow up of cohorts**	
Soni [15]	1	1	1	1	1	1	0	1	7
Assous [23]	1	1	1	1	1	1	0	1	7

Note: A study can be awarded a maximum of 1 point for each numbered item within the Selection and Exposure categories. A maximum of 2 points can be given for Comparability.

### Meta-analysis results

**Figure 2:**
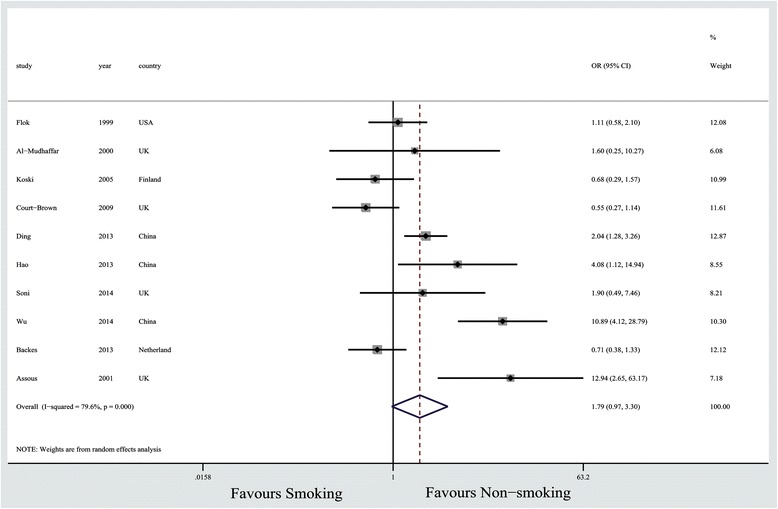
**Forest plot for the association between smoking and the risk of wound complications of DIACFs after ORIFs.**

A meta-analysis of pooled data was performed to analyze the potential risk factors for postoperative wound complications. Table 3 summarizes the pooled results. Bone graft (OR, 1.74; *p* = 0.007), diabetes (OR, 9.76; *p* < 0.00001), no drainage (OR, 5.68; *p* < 0.00001) and fracture severity (OR, 3.31; *p* < 0.00001) were the significant risk factors for postoperative wound complications of CDICFs after ORIFs. However, female patients, ORIFs performed within 14 days of injury, smoking (Figure 2), hypertension, and drinking did not make significant differences in the risk of wound complications (*p* > 0.05). In addition, ORIFs performed within 14 days of injury and smoking did not significantly affect the rate of major wound complications (*p* > 0.05) (Table 4).

**Table 3:** **Pooled results of overall wound complications for potential risk factors**

**Potential risk factors**	**No. of studies**	**Exposed group**	**Non-exposed group**	**Pooled OR**	**95% CI**	**P value**	**I** ^**2**^
Female	4	66/299	142/618	0.83	0.42-1.87	0.59	65%
Surgery timing (d) >14 (vs.≦14)	3	32/135	48/188	0.86	0.51-1.46	0.58	0%
Bone graft	4	54/252	117/730	1.74	1.17-2.59	0.007	49%
Smoking	10	180/681	169/963	1.90	0.97-3.30	0.06	80%
Hypertension	2	22/114	82/384	0.92	0.54-1.56	0.75	0%
Sanders classification (II.III/IV)	4	37/98	134/707	3.31	2.02-5.44	<0.00001	0%
Diabetes	4	20/33	94/367	9.97	4.43-23.50	<0.00001	0%
Drinking	2	26/107	48/142	0.85	0.19-3.76	0.83	55%
No drainage	2	63/268	15/162	5.68	2.91-11.09	<0.00001	0%
Female	4	66/299	142/618	0.83	0.42-1.87	0.59	65%

**Table 4:** **Pooled results of major wound complications for potential risk factors**

**Potential risk factors**	**No. of studies**	**Exposed group**	**Non-exposed group**	**Pooled OR**	**95% CI**	**P value**	**I** ^**2**^
Surgery timing (d) >14 (vs.≦14)	2	15/125	13/135	1.26	0.58-2.74	0.57	15%
Smoking	2	12/117	15/134	0.99	0.46-2.17	0.99	36%

**Figure 3:**
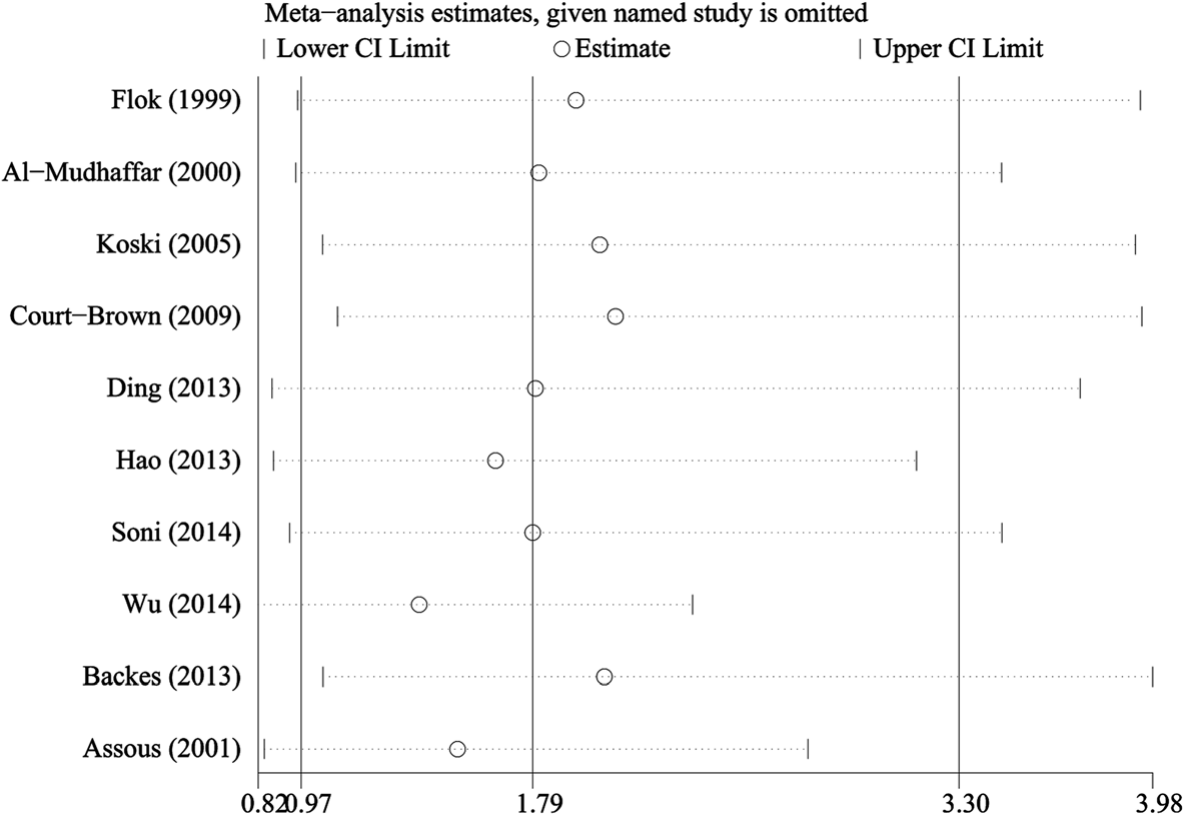
**Sensitivity analysis to examine the influence of each individual study on the overall OR of smoking.**

High heterogeneity was detected among studies evaluating as potential risk factors for smoking, female patients, and drinking (*I*^*2*^ > 50%). For smoking, sensitivity analysis was performed. The results demonstrated that the association was similar when one study was omitted at one time (Figure 3). Based on the various diversities (publication year, study design, study quality, region, operative approach, type of closure), we conducted meta-regression analysis to find the origin of the heterogeneity among studies, but the results demonstrated that these factors did not significantly influence the association between smoking and the risk of wound complications (Table 5). For female patients, we also performed sensitivity analysis. The result showed that once Backes et al.’s study [22] was excluded, the heterogeneity of the pooled results would drop from 65% to 3%. However, the pooled outcome was still similar (OR, 1.23; *p* > 0.05).

**Table 5:** **Meta-regression for variables that influence the association between smoking and risk of wound complications**

**Variables**	**Study variance (ţ** ^**2**^ **)**	**P value**	**Residual variation due to heterogeneity (I** ^**2**^ **)**
Publication year	1.04	0.75	80.85%
Study design	0.86	0.26	79.73%
Operative approach	1.06	0.89	80.70%
Type of closure	0.94	0.35	78.99%
Study quality	0.78	0.18	74.83%
Study region	0.88	0.26	78.71%

**Figure 4:**
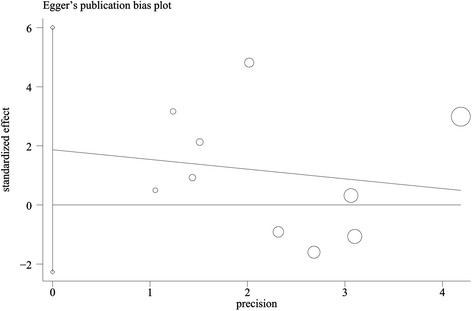
**Egger’s test to assess the publication bias.**

According to the results of Begg’s test (*p* = 0.474, continuity corrected) and Egger’s test (*p* = 0.328, Figure 4), there was no significant publication bias in this study.

## Discussion

The most important finding of the meta-analysis was that bone graft, diabetes, no drainage and fracture severity were identified as risk factors for wound complications of CDICFs after ORIFs, while ORIFs performed within 14 days of injury, smoking, hypertension, drinking and female patients made no significant differences.

### Patient-related risk factors

The results confirmed that fracture severity had a positive correlation on the postoperative wound complications in this study (OR = 3.31), which was consistent with previous results [21,22,27]. Backs et al. also reported that an American Society of Anesthesiologists (ASA) classification higher than ASA 1 was associated with an increased risk [22]. The disruption of microcirculation in the soft tissue could be a reason for it [31]. The more severe and complex fractures also increase the operative time that was associated with wound complications.

Our results showed that there was no significant difference in risk of postoperative wound complications between patients with or without a history of smoking, but a trend of more wound complications in patients with smoking history was observed (26.4% vs. 17.5%). Consistent results were observed in several studies [18,21,32,33]. Based on a recent systematic review that included 6356 patients with fractures, trends toward more superficial and deep infections of postoperative wounds in smokers were noted; however, the differences were also not significant [33]. Nicotine is a vasoconstrictor that reduces nutritional blood flow to the skin, resulting in tissue ischemia and impaired wound healing [16]. Moreover, it could increase platelet adhesiveness thereby raising the risk of thrombotic microvascular occlusion and tissue ischemia [34]. The subcutaneous synthesis of collagen (a critical determinant of the strength of an operative wound) was also impeded by smoking [35]. In addition, Moller et al. found that an effective smoking intervention program six-eight weeks before surgery reduces postoperative morbidity [36]. Based on results, we recommend that a smoking-cessation should be adopted before ORIFs.

Diabetes was the strongest risk factor for wound complications (OR = 9.97). Patients with diabetes had a 9.97-times greater risk of wound complications when compared with patients without diabetes. It has been demonstrated that diabetes could impede wound healing due to microvascular abnormalities [16].

As for female patients, drinking and hypertension, the results showed that they were not associated with the rate of wound complications. Similar results were reported in previous studies [21,22]. In our study, the average age of patients was relatively young (40.4 years old). The fractures in women generally were caused by relatively low-energy trauma that produced less severe injuries than those in men [37].

### Surgery-related risk factors

We found that bone grafting would increase the risk of wound complications (OR = 1.74). Similarly, a systematic review revealed that the infection rate in the bone graft group was higher than that in non-graft group (8.3% vs. 6.3%) [38]. Other studies also reported higher complications rate in the treatment of DICAFs with bone grafts [39,40]. Bone grafts in this meta-analysis included autogenous and allogenous bone grafts. Due to poor blood supply at the surgical site and serious soft tissue injury, the allogenous bone grafting with weak antibacterial properties might increase wound complications, such as infection and oozing. Meanwhile, for autogenous bone grafts, the procedure of bone-graft harvesting increased the operative time and blood loss. Additionally, the duration of surgery and blood loss was associated with wound complications [14,16]. According to a survey, the complication rates associated with harvesting iliac bone grafts can be as high as 40%, including chronic pain, haematoma formation, scarring, nerve injury, and wound problems [41]. Moreover, the medial wall of the CDICF was also fractured, proper filling of the space might be impossible, and the speed of healing did not warrant the extra risk associated with a graft. Several studies found no objective radiographic or functional benefit to the use of bone grafts in the operative treatment of CDICFs [28,42]. Given the extra surgical complications and the lack of any demonstrable extra benefit associated with bone grafting, we recommend that it should not be performed during ORIFs for CDICFs.

Our results showed that conditions with no drainage increased the risk of postoperative wound complications (OR = 5.68). A closed suction drain reduces the formation of haematomas thereby decreasing the likelihood of prolonged oozing from the wound, delayed wound healing or infection of the wound [43]. Evidence suggests it is accomplished through increasing blood circulation by angiogenesis, removing oedema, increasing granulation tissue formation, and decreasing bacteria counts [44,45]. Based on the data in a multi-centre prospective randomized clinical trial, there was a decreased incidence of wound dehiscence after high-risk fractures when patients have drainage applied to their surgical incision; the risk of infection was 1.9 times higher in control patients treated without drainage [46]. Meanwhile, consistent with several studies detected the similar result [47-49]. Nevertheless, drainage might also become contaminated and act as a conduit for infection, thus timely removals should be a concern.

For surgical timing, there is no difference between whether ORIFs were performed within 14 days of the injuries or later in our study. The correlation between surgical timing and wound complications remains an unsettled issue. Surgeries should be carried out after the condition of soft tissue improves [14]. Nevertheless, if ORIFs were postponed after complete regression of soft tissue oedema, haematoma would occur with the formation of a fibrous bony callus making separation and reduction of fracture fragments difficult, which would influence the final outcomes [50]. Wu et al. found that the risk of wound complications for ORIFs occurring within three days of injury was 5.47 times higher than that of surgeries delayed more than three days. Likewise, Al-Mudhaffar et al. suggest that it could be appropriate to delay ORIFs for at least seven to 10 days or until wrinkling of the skin reappears prior to afflicting a second assault on the soft tissue envelope for all calcaneal fractures requiring surgical interventions [19]. In contrast, Ho et al. reported that if ORIF was performed within 48 hours of injury by experienced orthopaedic trauma surgeons, fractures could be stabilized with relatively low rates of wound complications [51]. Meanwhile, Tennent et al. found that the infection rate increased significantly if the interval between injury and operation was greater than 14 days [32]. Therefore, for more objective facts, further high quality randomized controlled trials are required.

In this study, several risk factors were not combined due to inconsistent data forms or insufficient data. Longer operative time [14], prolonged tourniquet time [19], static skin distraction [14], a high number of persons present in the operating room [16], surgeon lacking surgical experience [21], higher body mass index [29] and a single-layer closure method [29] have earlier been identified as the risk factors associated with increased wound complications.

### Strengths and limitations

This study has several strengths. Firstly, to the best of our knowledge, this study is the first meta-analysis that quantitatively summarizes risk factors for wound complications of CDICFs after ORIFs. Secondly, the pooled outcomes are reliable due to our comprehensive data search, rigorous evaluation of methodological quality and the heterogeneity measure. Moreover, most included studies came from level-one trauma centers.

It is undeniable that there are some limitations in this study. First, all the included studies were retrospective cohort studies or case–control studies involving unavoidable recall and interviewer biases. Second, significant heterogeneity was detected in some pooled outcomes. However, after sensitivity analysis, meta-regression analysis and excluded publication bias, the outcomes are reliable. In addition, unadjusted confounding factors, such as the application of antibiotics, the experience of surgeons and the compliance of patients, which could influence the rate of wound complications, were not fully accounted for in several studies.

## Conclusions

Based on available evidences, bone graft, diabetes, no drainage and fracture severity were identified as risk factors for wound complications of CDICFs after ORIFs. Meanwhile, a smoking-cessation should be adopted before ORIFs. Prior to surgical treatment of CDICFs, patients who have the risk factors identified in this study should be counselled concerning the possible complications that might arise after ORIFs. Meanwhile, clinicians should consider the relevant risk factors to choose a better therapeutic strategy. In addition, future well-designed studies should be conducted to confirm these findings.

## Additional files

Additional file 1:
**Checklist S1 PRISMA checklist for this meta-analysis.**
Additional file 2:
**Search strategy for PubMed.**
Additional file 3:
**Search strategy for Embase.**

## Abbreviations

CI: Confidence interval
CDICF: Closed displaced intra-articular calcaneal fracture
ORIF: Open reduction and internal fixation
OR: Odds ratio
